# Gene Expression Analysis and Metabolite Profiling of Silymarin Biosynthesis during Milk Thistle (*Silybum marianum* (L.) Gaertn.) Fruit Ripening

**DOI:** 10.3390/ijms21134730

**Published:** 2020-07-02

**Authors:** Samantha Drouet, Duangjai Tungmunnithum, Éric Lainé, Christophe Hano

**Affiliations:** 1 Laboratoire de Biologie des Ligneux et des Grandes Cultures (LBLGC), INRAE USC1328, University of Orleans, 21 rue de Loigny la Bataille, F-28000 Chartres, France; samantha.drouet@univ-orleans.fr (S.D.); duangjai.tun@mahidol.ac.th (D.T.); eric.laine@univ-orleans.fr (É.L.); 2 Bioactifs et Cosmétiques, CNRS GDR3711, CEDEX 2, 45067 Orléans, France; 3 Department of Pharmaceutical Botany, Faculty of Pharmacy, Mahidol University, 447 Sri-Ayuthaya Road, Rajathevi, Bangkok 10400, Thailand

**Keywords:** abscisic acid, flavonolignans, fruit development, gene expression, metabolite profiling, *Silybum marianum*, silymarin

## Abstract

Mature fruits (i.e., achenes) of milk thistle (*Silybum marianum* (L.) Gaertn., Asteraceae) accumulate high amounts of silymarin (SILM), a complex mixture of bioactive flavonolignans deriving from taxifolin. Their biological activities in relation with human health promotion and disease prevention are well described. However, the conditions of their biosynthesis in planta are still obscure. To fill this gap, fruit development stages were first precisely defined to study the accumulation kinetics of SILM constituents during fruit ripening. The accumulation profiles of the SILM components during fruit maturation were determined using the LC-MS analysis of these defined developmental phases. The kinetics of phenylalanine ammonia-lyase (PAL), chalcone synthase (CHS) and peroxidase (POX) activities suggest in situ biosynthesis of SILM from l-Phenylalanine during fruit maturation rather than a transport of precursors to the achene. In particular, in contrast to laccase activity, POX activity was associated with the accumulation of silymarin, thus indicating a possible preferential involvement of peroxidase(s) in the oxidative coupling step leading to flavonolignans. Reference genes have been identified, selected and validated to allow accurate gene expression profiling of candidate biosynthetic genes (*PAL*, *CAD*, *CHS*, *F3H*, *F3’H* and *POX*) related to SILM accumulation. Gene expression profiles were correlated with SILM accumulation kinetic and preferential location in pericarp during *S. marianum* fruit maturation, reaching maximum biosynthesis when desiccation occurs, thus reinforcing the hypothesis of an in situ biosynthesis. This observation led us to consider the involvement of abscisic acid (ABA), a key phytohormone in the control of fruit ripening process. ABA accumulation timing and location during milk thistle fruit ripening appeared in line with a potential regulation of the SLIM accumulation. A possible transcriptional regulation of SILM biosynthesis by ABA was supported by the presence of ABA-responsive cis-acting elements in the promoter regions of the SILM biosynthetic genes studied. These results pave the way for a better understanding of the biosynthetic regulation of SILM during the maturation of *S. marianum* fruit and offer important insights to better control the production of these medicinally important compounds.

## 1. Introduction

**Figure 1:**
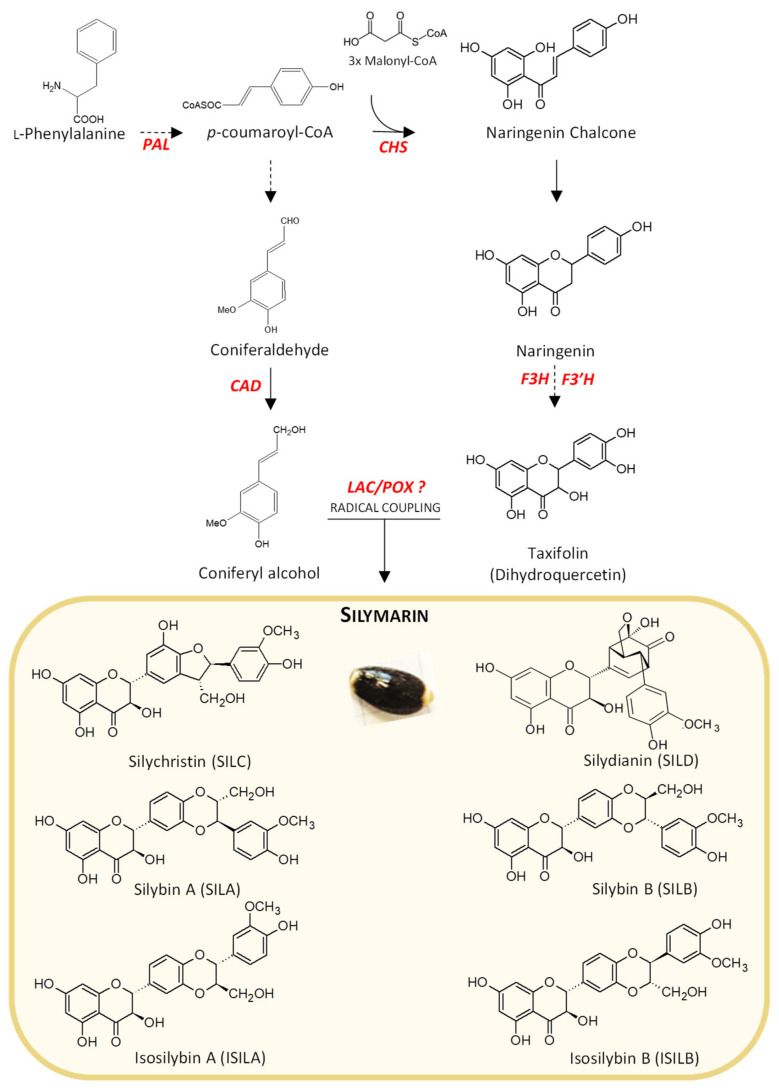
Partial scheme of the silymarin (SILM) flavonolignans biosynthesis pathway in *S. marianum*. Flavonolignans are mainly accumulated in mature achenes (yellow box). In red are presented genes potentially involved in this pathway: *PAL* (*l-phenylalanine ammonia-lyase*), *CAD* (*cinnamyl alcohol dehydrogenase*), *CHS* (*chalcone synthase*), *flavanone 3-dioxygenase* (*F3H*), *flavone 3′-hydroxylase* (*F3’H*), *LAC* (*laccases*) and *POX* (*peroxidases*). The dotted arrows indicate a single step, while dashed arrows indicate several steps in the metabolic pathway while the full arrows indicate direct synthesis of the compound through an enzyme.

*Silybum marianum* ((L.) Gaertn.) is an annual or biennial Asteraceae plant native to the Mediterranean region. It is currently widespread in Southern Europe, Western Asia, North Africa, Australia and North America. It is one of the oldest medicinal plants, already used in Greek and Roman medicines to help digestion or to treat liver and/or gallbladder disorders [1]. In the Middle Ages, its preparation extracts have been used to cure melancholy or black bile, both associated with various hepatic dysfunctions [2]. Nowadays, there is still a great deal of interest in this plant extract for medicinal and cosmetic applications, mainly due to the high accumulation in its fruits of the so-called silymarin (SILM), a complex mixture of flavonoids and flavonolignans (Figure 1). Its efficacy in the treatment of several diseases including liver disorders [1,3,4], inflammatory reactions [5,6] and oxidative stress protection [7,8,9,10] has been demonstrated. Most of these properties depended on the accumulation of silybins, a mixture of flavonolignan diastereoisomers [11].

The main flavonolignans isolated from milk thistle are derived from flavonoid taxifolin (TAX): silybin A (SILA), silybin B (SILB), isosilybin A (ISILA), isosilybin B (ISILB), silychristin (SILC), and silydianin (SILD) [11,12,13]. Other types of flavonolignans have been described in many plant species in the literature such as in *Oryza sativa* [14], *Onopordon corymbosum* [15], *Sasa veitchii* [16], *Lepidium meyenii* [17] or *Hydnocarpus anthelmintica* [18]. But milk thistle fruit remains, by far, the primary source of flavonolignans derived from the oxidative coupling of TAX and *E*-coniferyl alcohol and the unique SILM flavonolignans bioactive mixture.

In *S. marianum*, SILM flavonolignans are accumulated in the fruit (i.e., achene, sometimes incorrectly referred to as seed in the literature) [19]. Elucidation of biosynthetic steps and regulatory mechanisms leading to the production of SILM flavonolignans in milk thistle fruit is challenging as few genomic sequences from this species are available. Taking advantage of our current knowledge of the phenylpropanoid biosynthetic pathway in plants, it may be possible to propose a putative biosynthetic sequence leading to SILM flavonolignans and to point some key structural genes (Figure 1). The supposed biosynthetic sequence involved the deamination of l-Phe into trans-cinnamic acid by l-Phe ammonia-lyase (phenylalanine ammonia-lyase (PAL), EC 4.3.1.5), a branch-point enzyme between primary and secondary metabolism in plants. Trans-cinnamic acid is a crucial precursor to several phenylpropanoid derivatives including *E*-coniferyl alcohol in a metabolic sequence involving cinnamyl alcohol dehydrogenase (CAD, EC 1.1.1.195), and TAX in a metabolic sequence involving chalcone synthase (CHS, EC 2.3.1.74), flavanone 3-dioxygenase (F3H, EC 1.14.11.12) and flavone 3’-hydroxylase (F3’H, EC 1.14.13.88). Lastly, oxidative coupling reactions between TAX and E-coniferyl alcohol mediated by laccase(s) (LAC(s), EC 1.10.3.2) and/or peroxidase(s)(POX(s), EC 1.11.1.x) lead to different SILM flavonolignans (Figure 1). It is assumed that their biosynthesis resulting from the oxidative coupling between *E*-coniferyl alcohol and TAX could take place at this site of accumulation. The in vitro biochemical characterization of an ascorbate peroxidase enzyme (APX1) involved in the production of SILA/B and ISILA/B has been reported [20].

To date, however, no information is available on the production of other essential SILM flavonolignans, such as SILD and SILC. Biosynthesis of the latter has been proposed to involve a separate, and possibly more complex, oxidative coupling process that could infer the involvement of other POX or even LAC as well as of dirigent proteins [21,22], already described to direct the stereoselective biosynthesis of lignans in many plant species [23,24]. Besides, with the biosynthesis of *E*-coniferyl alcohol in the pericarp, a complex spatial organization has been suggested, with TAX biosynthesis suggested to be located in the flower [20], thus requiring the transport of this latter to the pericarp. However, this hypothesis is based on an interpretation of gene expression data limited to one single stage of fruit development. Therefore, a full-length analysis of the *S. marianum* fruit development process would be appropriate to check the validity of this hypothesis. To take a critical step towards better monitoring and understanding of SILM biosynthesis, it is therefore important to have a precise description of the various developmental stages during fruit maturation. Little attention has been paid to this point [25,26,27], and so little is known to date about the precise timing of accumulation of SILM during *S. marianum* fruit development. The lack of validation of reference genes for the study of gene expression in milk thistle is also an obstacle to the understanding of SILM biosynthesis regulation during achene development.

More detailed information on the spatiotemporal production of SILM flavonolignans, as well as on the regulation of their biosynthesis, could provide important information to optimize their accumulation. Both in the model plant *Arabidopsis thaliana* and crops, such as flax (*Linum usitatissimum* L.), it has been shown that abscisic acid (ABA) acts as a key phytohormone involved in the control of many aspects of seed development and phenylpropanoid biosynthesis. ABA has been shown to regulate several genes associated with fruit and seed maturation, and stress response including flavonoid and lignan biosynthesis [28,29,30]. Therefore, specific interest may be brought to the timing and position of ABA accumulation in *S. marianum* achene.

In the present study, six stages of development of *S. marianum* achenes were first adequately defined, enabling us to establish the precise timing and location SILM biosynthesis during fruit ripening. The enzymatic activities of PAL, CHS, LAC and POX during maturation were also determined in parallel. We identified reference genes from genomic data, selected and validated to follow accurately by RT-qPCR the spatiotemporal gene expression of candidate (i.e., *PAL*, *CAD*, *CHS*, *F3H*, *F3’H* and *POX*) genes potentially involved in flavonolignans biosynthesis. The linkage between gene expression, enzymatic activity, and accumulation of SILM was studied. Finally, ABA’s potential role was assessed by determining its accumulation profile and by analyzing the expression of genes involved both in its biosynthesis (*ABA1*) and signaling (*LEC2*).

## 2. Results and Discussion

### 2.1. Morphological Characterization of Milk Thistle Achene Development

**Figure 2:**
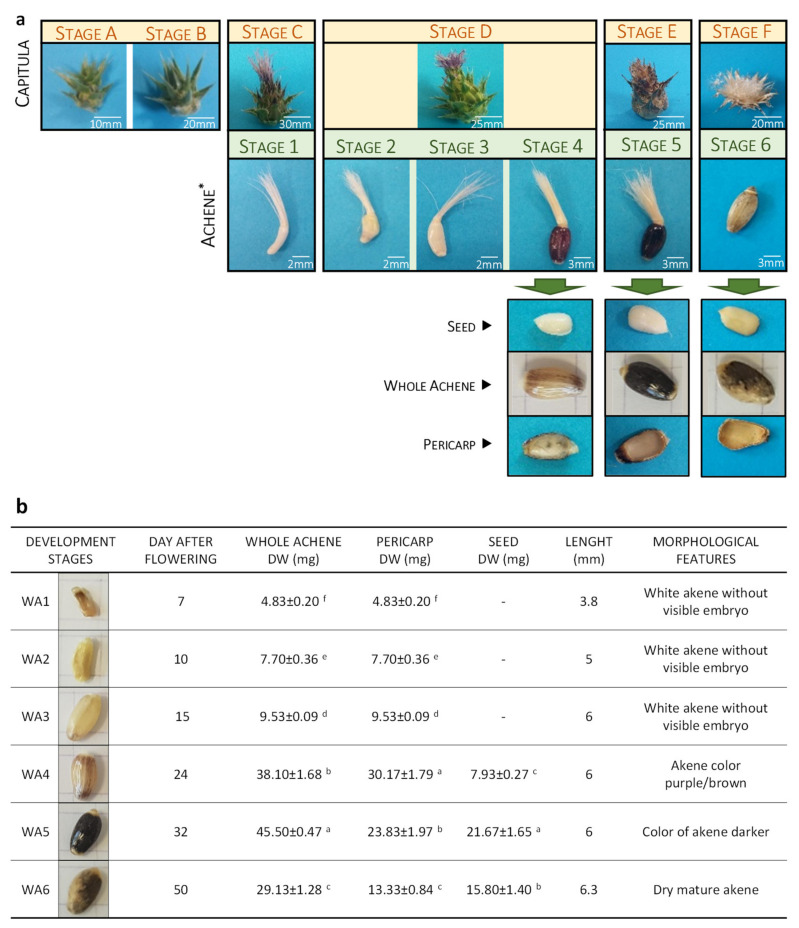
Development stages of the *S. marianum* achene defined according to their morphological characteristic. (**a**) Six achene developmental stages were defined. For the developmental stages 4, 5 and 6 achenes were manually dissected to allow the visualization of both seed and pericarp. The capitula morphology corresponding to each defined achene developmental stage is presented. Note that the capitula morphology is not predictive of the fruit developmental stage (see text for explanations). (**b**) Morphological features of achene maturation during time such as achene, seed, and pericarp length, dry weight (DW) and day of flowering (DAF). Each value represents means ± SD of *n* = 10 independent sampling. Different letters indicate significant differences at *p* < 0.05.

Fruit production stages have been described as one of the most critical processes in plant life. Such developmental stages may be defined as morphological features of a capitula or achene, or in days after anthesis (DAA) or flowering (DAF) [29]. However, since achene development may be affected by numerous factors including genetic factors (varieties / ecotypes) as well as environmental factors (e.g., light, temperature, and soil properties) and growing conditions (outdoor vs. greenhouse conditions), we decided to identify milk thistle fruit developmental stages according to achene morphological characteristics. Under greenhouse conditions, a complete development cycle leading to fully mature *S. marianum* fruits was achieved in about 50 days after flowering and six different developmental stages were described based on morphological characteristics of achene (Figure 2).

First, at stage 1 (DAF7), the achene was white / cream with no visible seed, the pericarp began to develop and accounted for the total weight of the achene. The fresh weight ratio (FW) to dry weight (DW) was high at this point, with a value around 10 (data not shown).

Subsequently, pericarp continued to develop gradually with stage 2 (DAF10) and stage 3 (DAF15), with a doubling of the FW value between stage 1 and stage 3 of development and the seed at stage 3 of development. At this stage of development, the pericarp color began to become pink. The ratio of FW to DW was still high.

At stage 4 (DAF24) the pericarp became purple and the white seed showed an active growth of around 20% of the achene weight. The ratio of FW to DW began to decrease, thus highlighting the transition from morphogenesis to maturation, with the achene having reached its final length.

At stage 5 (DAF32), the white seed accounted for about 50 % of the achene weight, the FW/DW ratio continued to decrease, confirming the start of fruit desiccation, and the achene color was dark brown.

Finally, stage 6 (DAF50) corresponded to the mature achene, with the light beige seed accounting for more than 55% of the achene weight. The observed decreases in DW up to the minimum values confirmed the completion of the desiccation. We also noted the lightening of pericarp as a result of the appearance of air bubbles in-between the most external cell layers of pericarp (Figure 2a).

The seed showed the presence of two cotyledons and has shown rapid growth from stage 3 to 6 of development, while the pericarp weight has gradually decreased from 30 to 13 mg from stage 4 to 6 of development. Note that the apparent capitula (flower head) morphology was also presented with the corresponding defined developmental stage in Figure 2a to facilitate sampling. For example, a capitula with a pink pappus (stage C) corresponding to the developmental stage 1 of fruit. Note, however, that capitula morphology is not predictive of the developmental stage of the fruit, since it is later centripetal and not completely synchronous. This further confirms the importance of precisely defining the stages of development and of breaking the capitula before the analysis is carried out.

In the literature, in addition to the mature stage, three further stages of maturation have been described in terms of the appearance of capitula (i.e., early flowering, mid-flowering with dry flowers, late flowering with dry flowers and dehiscence of capitula) [25,26,27], corresponding here to fruit colors ranging from white / cream, purple to (dark) brown. If these stages allow for a general view of maturation, they do not allow for a precise characterization of achene development. Here, six different achene ripening stages were precisely defined (Figure 2), thus simplifying the sampling method and increasing the accuracy of the molecular and (bio)chemical analysis of the tissue.

### 2.2. Accumulation Kinetic of SILM Constituents during S. marianum Fruit Development

**Figure 3:**
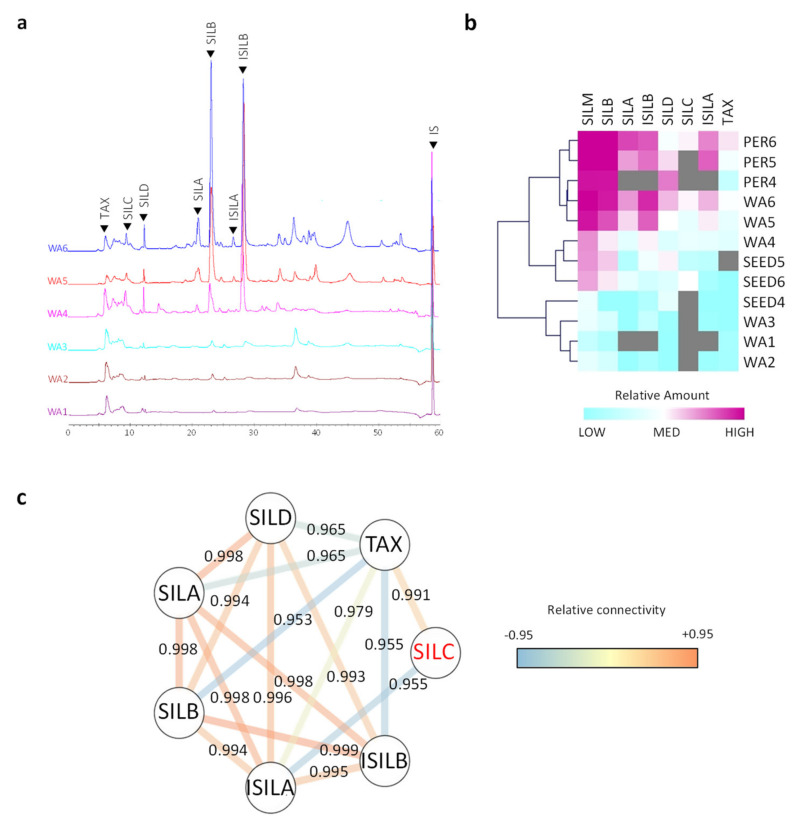
Accumulation of SILM compounds in *S. marianum* during achene development. (**a**) Chromatograms HPLC superposition of all 6 achene developmental stages showing the accumulation of SILM over time by comparison. IS: internal standard (6-methoxyflavone). (**b**) Extraction of compounds in whole achene (WA), pericarp (PER) and seed (SEED) represented in MeV (Multiple Experiment Viewer). Color scale is blue (weak content) to violet (high content) and grey color indicates not detected content. For quantitative values (referred to Table S1 expressed in mg/g DW). Values are means of *n* = 3 independent experiments; (**c**) the metabolite network was constructed using Cytoscape software 3.7, with a 0.95 cut-off value. Color edges from blue, yellow to red indicate increasing strength of the connection between the compounds.

Accumulation kinetics of SILM flavonolignans have been studied along the defined developmental stages of the fruit (Figure 3, Table S1). HPLC chromatograms revealed a major accumulation of flavonolignans during achene maturation starting from stage 4 of development (WA 4 (i.e., whole achene at developmental stage 4); Figure 3a, Table S1).

The accumulation of SILM increased dramatically at stage 4 when the desiccation process started. SILM enrichment was observed in pericarp compared to seed (Figure 3B, Table 1, Table S1).

SILM constituents have been quantified in whole achene (WA) for all defined developmental stages of the fruit and in manually separated pericarp and seed from developmental stages 4 to 6 (Figure 3b, Table S1). Accumulation kinetics of each SILM constituent also showed the same spatiotemporal location with accumulation starting from stage 4 of development with maximum values achieved in mature fruit (developmental stage 6) and mainly localized in pericarp. However, SILB, SILD and SILC also accounted for substantial amounts (over 1 mg/g DW) in the seed at the later stage of development. The presence of these three flavonolignans could indicate the potential transport of these molecules from the pericarp to the seed. Since milk thistle oil is rich in polyunsaturated fatty acids [31] that are more prone to oxidation, it can be assumed that the presence of these antioxidant compounds may contribute to the oxidative stability.

SILB and SILD were the first two flavonolignans to be detected in *S. marianum* fruit during its maturation process (Figure 3). In pericarp, these two compounds were also detected in high levels at stage 4 of development, while SILC was detected later (developmental stage 6) in high levels in the same tissue. This difference could suggest the involvement of different enzymes, more complex biosynthesis or a different regulation. Little is known about the biosynthetic sequence leading to the biosynthesis of SILM flavonolignan. The main hypothesis concerning milk thistle flavonolignans biosynthesis suggested an oxidative coupling between TAX and *E*-coniferyl alcohol. In recent years, it has been proposed that the involvement of dirigent proteins should explain the preferential accumulation of some SILM components [22,32]. The Metabolite Network was proposed to evaluate the biochemical connectivity between the various intermediates and/or the branch of a biosynthetic pathway [33]. Except for SILD, the metabolite network showed a strong biochemical connectivity between each compound (Figure 3c). According to this network, SILD only has a high connectivity with TAX (its precursor) and ISILA. It has been proposed that a strong connectivity between the substrate and the product of a considered enzymatic step suggests a weak contribution of this step to the flux control of this biosynthetic pathway and a simpler regulation [33]. These results could, therefore, suggest that there are at least two different regulations for this biosynthetic pathway during *S. marianum* fruit maturation.

### 2.3. Kinetic Study of Selected Enzymatic Activities Related to SILM Biosynthesis

**Figure 4:**
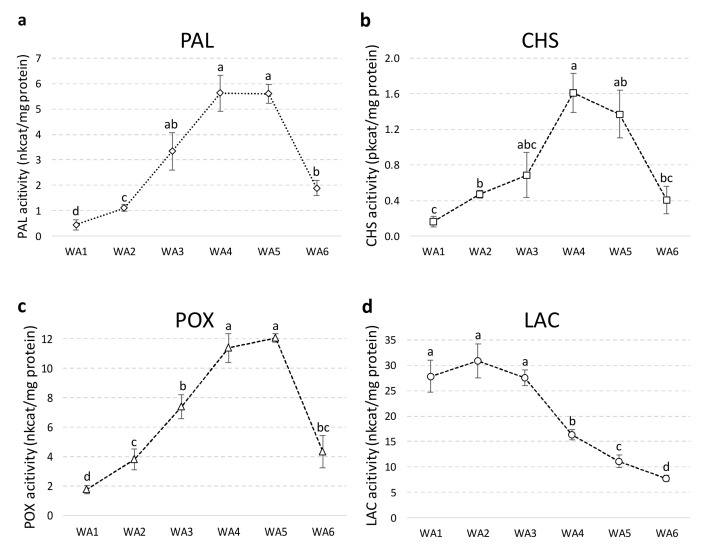
Time course evaluation during fruit development of specific phenylalanine ammonia-lyase (PAL) (**a**), chalcone synthase (CHS) (**b**), peroxidase (POX) (**c**) and laccase (LAC) (**d**) enzymatic activities in the soluble protein fraction from in whole achene of *S. marianum*. Values are the mean ± SD of 3 independent measurements. Different letters indicate significant differences at *p* < 0.05.

To gain a deeper insight into the timing of SILM biosynthesis and to discriminate between in situ production or transport, we then determined the activity of PAL, CHS, POX and LAC enzymes during fruit ripening (Figure 4).

PAL, CHS and POX enzymes showed a similar pattern of activity reaching maximum values at stages 4 and 5 before decreasing in mature fruit (Figure 4a–c). In sharp contrast, LAC activity changed independently of the other enzymes and was high at the early stages of development (Figure 4d). The developmental changes observed in their enzyme activities allow these enzymes to be grouped into two groups that reflect their possible involvement in SILM biosynthesis in *S. marianum*. High and significant correlations between PAL, CHS and POX were calculated, while LAC was not associated with any of these enzymes (Table S2).

The timing of PAL, CHS and POX activities is consistent with SILM accumulation during fruit maturation and could support the involvement of these enzymes in the in situ biosynthesis of SILM flavonolignans. Supportively, the biochemical characterization of one peroxidase active for the formation of SILB, but inactive for the formation of the other *S. marianum* flavonolignans, was presented [20]. Here, the results also favor the involvement of POX in flavonolignan biosynthesis rather than LAC in the final oxidative coupling step. However, the complete sequence of fruit development has been considered, and therefore this hypothesis is further reinforced. *PAL* gene expression has been reported in *S. marianum* fruit at a single stage corresponding to SILM accumulation [20]. Here, the detection of PAL activity indicates that this expression of this gene effectively leads to the production of a functional protein. It also shows that the phenylpropanoid pathway is (at least the first limiting step) active in situ at the time of SILM accumulation. We determined a similar spatio-temporal pattern for CHS activity. Likewise, at three developmental stages based on capitula morphology, Torres and Corchete [25] observed a *CHS* gene expression with a similar timing in *S. marianum*. The two CHS isoforms were also observed in various *S. marianum* organs, including one expressed in the pericarp, following our enzymatic assays. This suggests that the first step of flavonoid biosynthesis, during fruit maturation, is therefore also active in the pericarp. However, Lv et al. [20] suggested the hypothesis for the biosynthesis of the two SILM precursors, *E*-coniferyl alcohol and TAX, of a distinct spatial organization. This hypothesis was based on the analysis of RNAseq data from a single stage of immature fruit development (pericarp vs. seed), collected from outdoor plants 10 days after flowering, and by comparison with root, stem, leaf and flower conditions. First, in situ biosynthesis in the pericarp, at this single stage, of *E*-coniferyl alcohol was supported by the expression detected of several genes involved in its biosynthesis proposed from this study [20]. By contrast, ex situ TAX biosynthesis was proposed because only CHS gene expression was detected in that tissue, while several expressions of several biosynthetic genes (including *F3H* and *F3’H*) were detected in flowers [20]. This has led Lv et al. [20] to propose a separate spatio-temporal organization for the production of the two SILM precursors, including transportation of TAX from petals to pericarp. In sharp contrast, both Torres and Corchete [25] detected both *F3H* and *F3’H* gene expression in immature *S. marianum* fruits at 3 stages of development based on capitula morphology. Moreover, in contrast to this transport hypothesis, previous work observed flavonoid transport in seed was rather limited to intracellular movements between cytoplasm and vacuole, while symplastic interorgan transport was limited to basipetal movement [34,35]. To clarify this discrepancy, taking advantage of these defined developmental stages, our next step was to study the expression time course of SILM biosynthetic genes by RT-qPCR.

### 2.4. Expression of Genes Involved in Phenolic Compounds Synthesis

#### 2.4.1. Validation of Reference Genes

**Figure 5:**
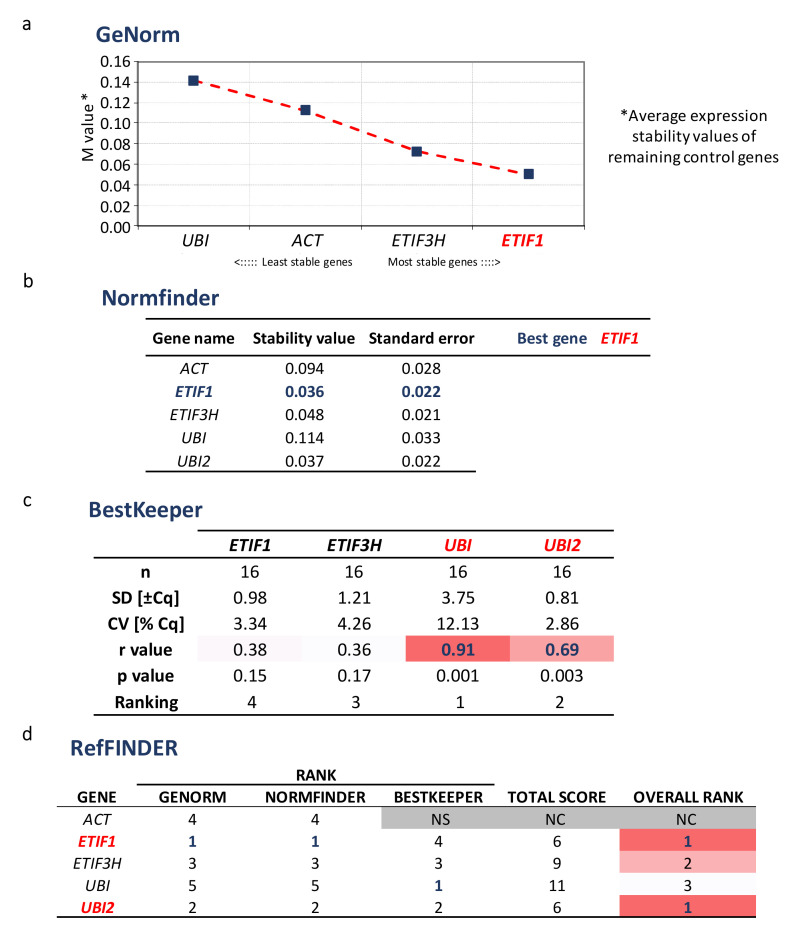
Gene expression stability analysis of the candidate reference genes for RT-qPCR gene expression study in *S. marianum* during fruit maturation according to GeNorm (**a**), NormFinder (**b**), BestKeeper (**c**) as well as RefFINDER ranking result (**d**). NS: not considered as stable enough by the software analysis. NC (Grey): not ranked. Figure 5c and d are represented as heatmap from white (lower stability) to pink (medium stability) and red (higher stability).

Before the gene expression analysis of selected biosynthetic genes, validation of reference genes is an essential prerequisite. A preliminary study of the gene expression of "housekeeping" genes should be carried out systematically in the tissues and experimental conditions studied to confirm their stability and to avoid any bias in the results [29,36,37,38]. Validation of reference genes is a very challenging step in the maturation of seeds and fruits [39]. Therefore, the first consisted in the identification candidate for the selection of reference genes. As a result, we identified 12 candidates in the genome of *S. marianum*. The characteristics of these genes are described in Table S3. The candidate genes not detected under all experimental conditions corresponding to the defined developmental stages have been excluded (Figure S1). Then, we evaluated the remaining selected reference genes using a variety of software (RefFinder, BestKeeper, GeNorm and Normfinder) that allowed us to study and classify their gene expression stability (Figure 5).

From this validation analysis, the two most stable reference genes to normalize the expression SILM candidate genes were *UBI2* and *ETIF1*.

#### 2.4.2. Gene Expression Analysis of Candidate Genes

**Figure 6:**
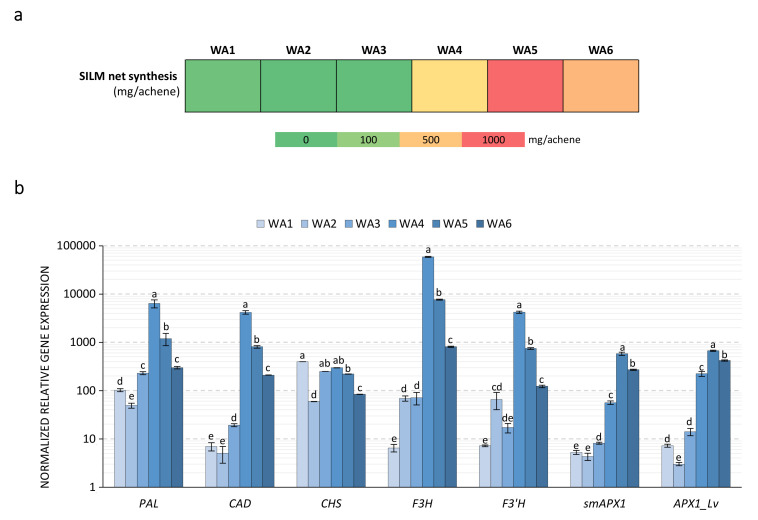
Kinetics of SILM synthetic gene expression during milk thistle achene maturation. (**a**) Kinetics of SILM net production (expressed in mg/achene) at each developmental stage in whole achene (WA) during milk thistle fruit maturation (calculated from Table 1). (**b**) Relative quantification using RT-qPCR of RNA expression of putative genes involved in *S. marianum* flavonolignans biosynthesis (*PAL*, *CAD*, *CHS*, *F3H*, *F3’H*, *smAPX1* and *APX1_Lv*). Values are the mean ± SD of 3 independent measurements. Different letters indicate significant differences at *p* < 0.05.

In *S. marianum*, as in other accumulating plant species, little is known about the regulation of flavonolignans biosynthesis. It is accepted that in *S. marianum*, flavonolignans biosynthesis implies the involvement of different branches of the phenylpropanoid biosynthetic pathway: the general branch leading to *p*-coumaroyl-CoA, from which two specific branches may originate: the monolignol pathway from which the *E*-coniferyl alcohol precursor is produced, and the flavonoid pathway from which the TAX precursor is produced (Figure 1). A final oxidative coupling step occurred between these two precursor moieties, leading to the different SILM flavonolignans. The complete coding sequences of the *PAL*, *CAD*, *CHS*, *F3H*, *F3’H* and *POX* genes were retrieved from the *S. marianum* genomic data to account for each of these metabolic branches leading to SILM flavonolignans. Their characteristics and comparison with the *A. thaliana* orthologous genes [40] are shown in Table S4. Their expression profiles during the development of *S. marianum* fruit established by RT-qPCR are shown in Figure 6.

In agreement with SILM production during fruit maturation (Table 1 and Figure 6a), all genes had a similar pattern of expression with a strong increase in their steady state mRNA levels reaching maximum values at stage 4 before decreasing as with fruit ripening (Figure 6b). These results are consistent with those presented by Torres and Corchete [25]; therefore, they are in favor of the complete in situ biosynthesis of SILM flavonolignans and their precursors. By comparison, Lv et al. [20] did not detect *F3H* and *F3’H* mRNA in immature fruit (10 DAF) using RNAseq as compared to other green vegetative tissue analyzed. This discrepancy may be explained by the difficulty of extracting high-quality RNA and/or proteins from seed tissue as shown by the difficulty of obtaining stable reference genes for this tissue [29,39,41,42]. The expression profile of the ascorbate peroxidase enzyme (APX1) is also consistent with this in vitro biochemical characterization, which showed its ability to synthesize both SILA/B and ISILA/B by Lv et al. [20]. Here, a second *POX* gene, different form the one previously identified and biochemically characterized (Figure S2), was identified from genomic data and its expression profile was consistent with the possible involvement in the biosynthesis of SILC and/or SILD and its derivatives. In addition to the action of oxidase, a more complex stereoselective sequence involving dirigent proteins (DIRs) has been suggested for the biosynthesis of these compounds [21,22]. The presence of different DIRs, for example, is responsible for the stereoselective accumulation of lignans in flax [24,43]. Future work on its biochemical characterization as well as the possible involvement of DIRs to be identified will be undertaken.

### 2.5. Relationship between Compounds and Kinetics of ABA Content

**Figure 7:**
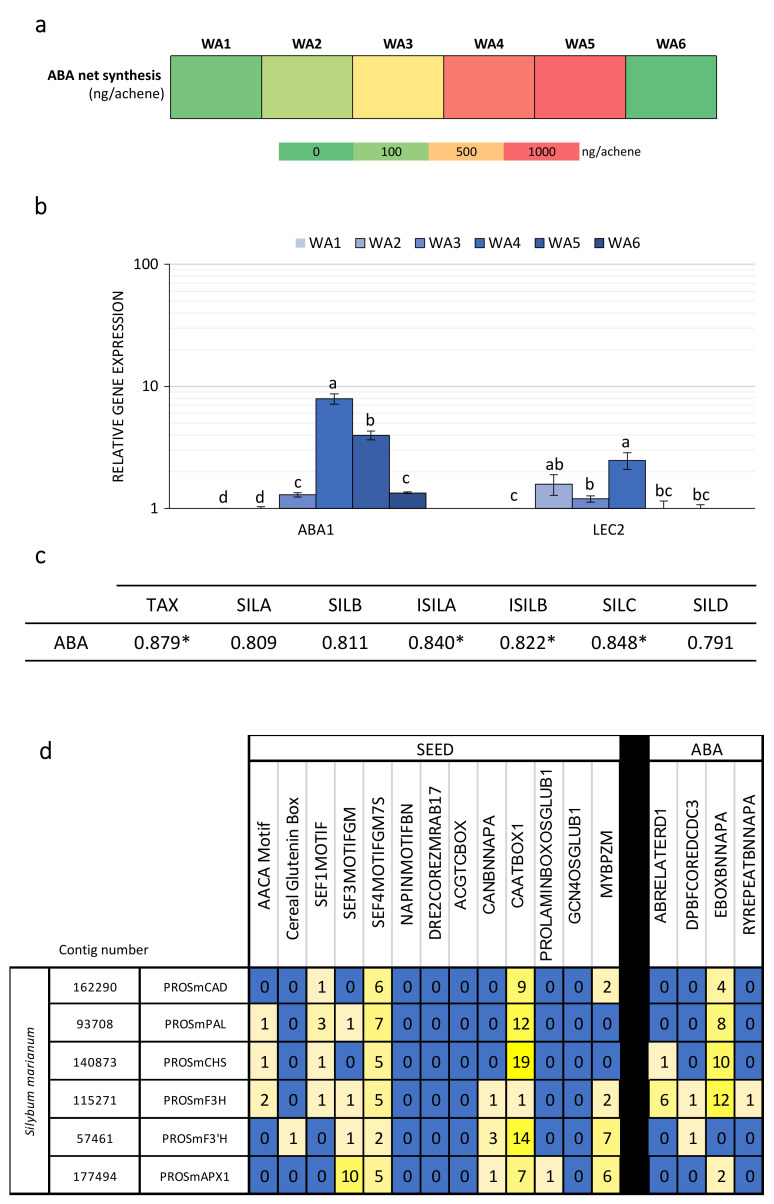
Linkage between compounds and ABA in whole achene stages. (**a**) Kinetics of abscisic acid (ABA) net production (expressed in ng/achene) at each developmental stage in whole achene (WA) during milk thistle fruit maturation (calculated from Table 1). (**b**) Relative quantification using RT-qPCR of RNA expression of 2 genes implicated in ABA flavonolignans biosynthesis (*ABA1* and *LEC2*). (**c**) Pearson correlation of extraction compounds in whole achene stages were performed by PAST software. (* *p* < 0.05, *n* = 3). (**d**) Identification of ABA-responsive cis-acting elements located in gene promoter gene sequences (from blue to yellow indicating low to high number of each cis-acting element). Values are the mean ± SD of 3 independent measurements. Characteristics of cis-acting elements are provided in Table S5. Different letters indicate significant differences at *p* < 0.05.

Many genes associated with the maturation of seeds and fruits or the biosynthesis of phenylpropanoids are regulated by ABA [30]. ABA quantification showed a significant increase during fruit maturation with important accumulation at stage 4 (Figure 7a).

Maximum production of ABA coincided with the preceding stage of the embryogenesis, but also observed increases in biosynthetic gene expression and enzyme activity leading to SILM flavonolignan production (Figure 2, Figure 3 and Figure 4, Figure 6). RT-qPCR analysis of the expression of genes involved in ABA biosynthesis (*ABA1*) and signaling (*LEC2*) confirmed this accumulation time course (Figure 7b). We observed significant associations between ABA accumulation and SILM biosynthesis (Figure 7c). ABA was detected both in the pericarp and in the seed (Table S1). According to these results, it appeared that the biosynthesis of flavonolignans could be regulated by ABA, with the seed appearance as a signal for the start of their biosynthesis. Such a regulation has already been proposed in the regulation of lignan biosynthesis in flax, in which seed development was needed for biosynthesis, and ABA acts as a key regulator [28,41]. Similarly, as observed in flax, in aborted *S. marianum* achenes, no active flavonolignan biosynthesis has been observed in the absence of seed development (data not shown). The potential contribution of ABA in the transcriptional regulation of SILM biosynthesis was further verified by in silico identification of putative ABA-responsive and fruit/seed-specific cis-acting elements (Figure 7d; Table S5). ABA is a central phytohormone involved in seed and fruit maturing regulation in many species of model and crop plants, such as *A. thaliana* and flax [28,29,30,44]. It has also been related to transcriptional regulation of biosynthesis of phenylpropanoids in seeds and fruits [28,29,30]. Here, information on the timing and location of ABA accumulation during *S. marianum* fruit maturation and its association with the expression of biosynthetic genes and SILM accumulation is of particular interest to understand how this metabolic pathway is regulated. Future work should be carried out to identify the transcription factors involved in this regulation and to characterize them functionally.

## 3. Materials and Methods

### 3.1. Plant Materials

The plants were grown in pots (30 cm in diameter and 30 cm in depth), packed with commercial garden soil (composition: 250 g/m^3^ N, 120 g/m^3^ P_2_O_5_, 80 g/m^3^ K_2_O, dry matter: 37%, organic matter: 65%, pH: 6.2, conductivity: 49 mS/cm, water retention capacity: 70% volume) in a phytotronic room at 25 °C under a 16-h photoperiod (30 µmol/m^2^/s total amount of photosynthetically active radiation) and relative humidity (RH) was around 30%. Plants were irrigated once a day using overhead mist irrigation, and one full watering per week until the full development cycle.

### 3.2. Chemicals

Solvents and reagents used in the present study were all of analytical grade or highest available purity (Fisher Scientific, Illkirch, France). Deionized ultrapure water was produced using a Milli-Q water-purification system (Millipore, Molsheim, France). All analytical solutions were filtered through 0.45 µm nylon syringe membranes prior to use. Commercial standards of TAX, SILC, SILD, SILA, SILB, ISILA and ISILB were purchased from Sigma-Aldrich (Saint-Quentin Fallavier, France).

### 3.3. Phytochemicals Analysis

Ultrasonic extractions (3 biological and 2 technical replicates) were performed using 60 mg (DW) of achene, pericarp or seed in 1 mL of 50% (*v*/*v*) aqueous ethanol as described by Drouet et al. [45]. For this purpose, an USC1200TH ultrasonic bath with the following inner dimension was used: 300 mm × 240 mm × 200 mm (VWR International, Fontenay-sous-Bois, France). Silymarin composition and quantity were determined by LC-MS using a Water 2695 Alliance (Waters-Micromass, Manchester, UK) coupled with a single quadrupole mass spectrometer ZQ (Waters-Micromass, Manchester, UK) as described previously [22,32].

### 3.4. Enzymatic Activities

#### 3.4.1. Total Soluble Proteins Extraction and Quantification

From 150 mg of fresh frozen (−80 °C) tissue, total soluble proteins were extracted by homogenization in 3 mL 0.1 M sodium borate buffer (SBB) pH 8.8 containing 10 mM β-mercaptoethanol as described by Hano et al. [46]. Protein concentration was measured with the Quant-iT Protein Assay Kit and Qubit^®^ 3.0 fluorometer according to manufacturer instructions (Thermo Scientific, Courtaboeuf, France).

#### 3.4.2. PAL Activity

PAL activity was spectrometrically determined, monitoring the formation of trans-cinnamate at 290 nm as described by Hano et al. [46].

#### 3.4.3. CHS Activity

CHS activity was determined by HPLC as described by Sun et al. [47], using *p*-coumaroyl-CoA, synthesized according to Beuerle and Pichersky [48], and malonyl-CoA (Sigma-Aldrich, Saint-Quentin Fallavier, France) as substrate, by monitoring at 289 nm the formation of naringenin from the subsequent non-enzymatic conversion of the formed naringenin by CHS activity.

#### 3.4.4. POX Activity

POX (peroxidase) activity was determined spectrometrically using guaiacol (Sigma-Aldrich, Saint-Quentin Fallavier, France) as substrate, and following the absorbance increase at 470 nm as described by Morawski et al. [49].

#### 3.4.5. LAC Activity

LAC (laccase) activity was determined spectrometrically at 415 nm, following the ABTS (2,2′-azino-bis(3-ethylbenzothiazoline-6-sulfonate) (Sigma-Aldrich, Saint-Quentin Fallavier, France) oxidation as described by Wang et al. [50].

### 3.5. Gene Identification

Gene identification by tBLASTn analysis on NCBI server using publicly available sequence contigs, generated from Illumina Hiseq data of *S. marianum* (NCBI:txid92921, WGS:LMWD01000001:LMWD01258575) using *A. thaliana* orthologs as queries with the comparison matrix BLOSUM62 (at the score value of > 300 and *e*-value < e−100). The results of these searches are presented in Table S3 (reference genes) and Table S4 (biosynthetic genes).

### 3.6. Gene Promoter Analysis

The corresponding putative promoter sequences were determined as the 1500 base pairs upstream of the predicted starting translation codons. Putative promoter sequences were submitted to PLACE [51] and PlantPAN2.0 [52] analyses to identify putative cis-acting regulatory DNA elements involved in seed expression and/or response to ABA.

### 3.7. RNA Extraction

The total RNAs of achene, pericarp and seed were extracted from crushed tissue in liquid nitrogen using the GeneJET Plant RNA Purification kit (Thermo Fisher Scientific, Courtaboeuf, France) following manufacturer’s recommendations. An additional DNase I treatment (RNase-free DNase, Qiagen, Courtabeauf, France) was applied directly to the column for 15 minutes at 25 °C to remove traces of contaminating DNA. Total RNAs were then quantified using a fluorometer and the QuantiT RNA Assay Kit (Life Technologies, Courtaboeuf, France) and Qubit fluorometer (Life Technologies, Courtaboeuf, France) according to the manufacturer’s instructions. RNA was then stored at −80 °C.

### 3.8. RT-qPCR Analysis

The first strand of cDNA was retro-synthesized from 50 ng of total RNA using the Maxima Reverse Transcriptase kit (Life Technologies, Courtaboeuf, France) according to manufacturer’s instructions and were stored at −25 °C. Quantitative PCRs were realized in 96-well plates using the PikoReal real time PCR system (ThermoFisher, Courtaboeuf, France) and DyNAmoColorFlash SYBR Green qPCR Kit (ThermoFisher, Courtaboeuf, France). Each reaction was performed as described in Corbin et al. [24]. Analysis of the data was performed with Pikoreal software. Three biological replicates and two technical repetitions were realized for each sample. Relative transcript levels were obtained using specific primers (Tables S3 and S4), designed with Primer3 software [53], and normalized using the comparative ΔΔCq method using two validated housekeeping reference genes.

### 3.9. Validation of Reference Genes

The evaluation of twelve candidate reference genes was performed with RefFinder, a web-based comprehensive tool developed for the evaluation, screening and selection of reference genes from extensive experimental datasets. RefFinder integrates the major available computational programs geNorm [54], Normfinder [38], BestKeeper [55] to compare and rank the tested candidate reference genes. Based on the rankings from each program, it assigns an appropriate weight to an individual gene and calculates the geometric mean for the overall final ranking [56].

### 3.10. ABA Extraction and Quantification

ABA extraction from developing milk thistle fruit was based on the procedure described by Renouard et al. [28]. Freeze-dried developing achenes (100 mg FW) were extracted for 16 h at 4 °C in the dark with MilliQ water (water/tissue ratio 50:1, *v*/*w*). ABA was quantified by ELISA assay Phytodetek ABA ELISA kit (Agdia EMA, Evry, France) using (±) cis–trans ABA (Sigma, Saint-Quentin Fallavier, France) as a standard. Experiments were realized in triplicates.

### 3.11. Statistical and Treatment of Data

At least three independent biological repetitions were performed to allow calculation of means and standard deviation. Boxplots were conducted using RStudio. The correlation matrix was obtained with PAST software by performing the Pearson parametric correlation test. Heat maps were produced using the MeV software computed with a hierarchical clustering analysis (HCA) representing the Euclidean distance with a clustering method with a complete linkage clustering. The metabolite network was visualized using the Cytoscape 2.8.3 software by representing only the significant Pearson Correlation Coefficient (PCC) values at *p* < 0.05 with a cut-off value of 0.60 (significant positive (in red) and negative (in blue) correlations). Colors from yellow to red indicate increasing PCC values and the connection size indicates the strength of the connection.

## 4. Conclusions

Silymarin (SILM) is a complex mixture of bioactive flavonolignans that accumulate milk thistle (*Silybum marianum* (L.) Gaertn., *Asteraceae*) in its mature achene fruits. These compounds are well known for their relationship to promote human health and prevent disease, but the conditions of their biosynthesis in planta remain elusive. Development stages of fruit were precisely described to study the kinetics of accumulation of SILM constituents during fruit ripening. During fruit maturation, the accumulation profiles of the SILM components were evaluated by LC-MS analysis at each of the development stages identified. Reference genes have been identified, selected and validated to allow accurate gene expression profiling of candidate biosynthetic genes. Enzyme activity and biosynthetic gene expression indicated a possible in situ biosynthesis of SILM from l-Phe during fruit ripening. The gene expression profiles were well correlated with SILM kinetic accumulation and preferential location in pericarp during *S. marianum* fruit maturation, reaching maximum biosynthesis when desiccation occurs. This observation led us to consider the possible involvement of abscisic acid (ABA), a key phytohormone in fruit ripening control, for which accumulation timing and location during fruit ripening were consistent with the potential regulation of the SLIM accumulation. This possible transcriptional regulation of SILM biosynthesis by ABA was further supported by the presence of ABA-responsive cis-acting elements in the SILM biosynthetic gene promoter regions studied. These results pave the way for a better understanding of the biosynthetic regulation of SILM during the maturation of *S. marianum* fruit, thereby providing important insights to better control the production of these medicinally important compounds.

## Figures and Tables

**Table 1:** Evaluation of SILM and abscisic acid (ABA) contents in whole achenes of *S. marianum* during achene maturation expressed per g DW as well as per achene.

Metabolite	WA1	WA2	WA3	WA4	WA5	WA6
SILM (mg/g DW)	0.14 ± 0.04 ^e^	0.42 ± 0.03 ^d^	0.50 ± 0.04 ^d^	4.58 ± 0.33 ^c^	24.20 ± 2.12 ^b^	52.46 ± 2.73 ^a^
SILM (mg/achene)	0.70 ± 0.21 ^e^	3.23 ± 0.23 ^d^	4.78 ± 0.36 ^d^	174.68 ± 12.46 ^c^	1100.97 ± 96.55 ^b^	1528.14 ± 79.59 ^a^
ABA (ng/g DW)	1.83 ± 0.38 ^e^	5.57 ± 0.63 ^d^	13.63 ± 1.42 ^c^	29.10 ± 1.91 ^b^	48.57 ± 1.56 ^a^	46.37 ± 2.15 ^a^
ABA (ng/achene)	8.86 ± 1.82 ^f^	42.86 ± 4.88 ^e^	129.97 ± 13.53 ^d^	1108.71 ± 72.69 ^c^	2209.78 ± 70.99 ^a^	1350.66 ± 62.60 ^b^

Each value represents means ± SD of at least *n* = 3 independent sampling. Different letters indicate significant differences at *p* < 0.05.

## Supplementary Materials

The following are available online at https://www.mdpi.com/1422-0067/21/13/4730/s1, Figure S1. a. Variation of Ct values for each of the 12 analyzed potential reference genes in whole achenes, pericarps and seeds during the 6 developmental stages of *S. marianum* maturation. b. Agarose gel electrophoresis analysis of amplified RT-qPCR fragments for each 12 analyzed potential reference genes (here analyzed in whole achenes of *S. marianum* at developmental stage 4); Figure S2. Alignment of APX_1Lv (from Lv., 2017) and smAPX (predicted in Augustus software) in Clustal omega; Table S1: Evolution of the accumulation of SILM and its different constituents during *S. marianum* fruit maturation (in whole achenes (WA), pericarps (P) and seed (S) from stage 1 to stage 6 of maturation); Table S2: Pearson correlation coefficient between PAL, CHS, POX and LAC activities determined during *S. marianum*; Table S2: Primers and characteristics of the genes used for gene references selection for RT-qPCR analysis in maturing fruit of *Silybum marianum*; Table S4: Primers and characteristics of the SILM biosynthetic genes and ABA biosynthetic and signaling genes used for gene expression by RT-qPCR analysis in maturing fruit of *Silybum marianum*; Table S5: List of cis-acting elements located in the SILM biosynthetic gene promoter regions.
