# Supplementary material for: Gene Expression Analysis and Metabolite Profiling of Silymarin Biosynthesis during Milk Thistle (Silybum marianum (L.) Gaertn.) Fruit Ripening
Source: Int J Mol Sci. 2020 Jul 2;21(13):4730. doi: 10.3390/ijms21134730 (PMC7370286; doi:10.3390/ijms21134730)
Supplement: Supplementary file 1 [file ijms-21-04730-s001.pdf]

SUPPLEMENTARY MATERIALS FOR

Gene Expression Analysis and Metabolite Profiling of Silymarin Biosynthesis during Milk Thistle (*Silybum marianum* (L.) Gaertn.) Fruit Ripening

Samantha Drouet[1,2], Duangjai Tungmunnithum[1,2,3], Éric Lainé[1,2] and Christophe Hano[1,2,*]

[1] Laboratoire de Biologie des Ligneux et des Grandes Cultures (LBLGC), INRAE USC1328, University of Orleans, 21 rue de Loigny la Bataille, F-28000 Chartres, France; samantha.drouet@univ-orleans.fr (S.D.); eric.laine@univ-orleans.fr (E.L.); hano@univ-orleans.fr (C.H.)

[2] Bioactifs et Cosmétiques, CNRS GDR3711, 45067 Orléans Cedex 2, France;

[3] Department of Pharmaceutical Botany, Faculty of Pharmacy, Mahidol University, 447 Sri-Ayuthaya Road, Rajathevi, Bangkok 10400, Thailand; duangjai.tun@mahidol.ac.th (D.T.)

* Correspondence: hano@univ-orleans.fr; Tel.: +33-237-309-753; Fax: +33-237-910-863 (C.H.)

Figure S1. a. Variation of Ct values for each of the 12 analyzed potential reference genes in whole achenes, pericarps and embryos during the 6 developmental stages of *S. marianum* maturation. **b.** Agarose gel electrophoresis analysis of amplified RT-qPCR fragments for each 12 analyzed potential reference genes (here analyzed in whole achenes of *S. marianum* at developmental stage 4).

a

b

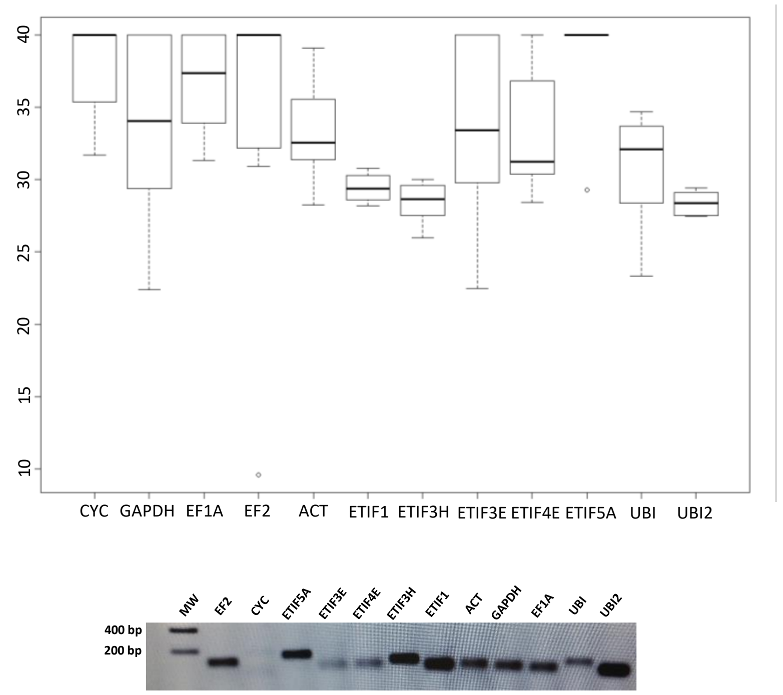

Figure S2. Alignment of APX_1Lv (from Lv., 2017) and smAPX (predicted in Augustus software) in Clustal omega

```
APX1_Lv    MAMPVVDTEYLKEIDKARRELRAFISNKNCAPIMLRLAWHDAGTYDVNTKTGGPNGSIRT  60
smAPX1     --MPVVDAEYLKEIETARRDLRAFISSKKCAPIMLRLAWHDAGTYDATTKTGGPNGSIRN  58
             *****:******:.***:******.*:*****************..***********.

APX1_Lv    EEEYSHGSNNGLKIAIDFCEEIKSKHPRITYADLYQLAGVVAVEVTGGPTVDFVPGRKDS  120
smAPX1     EEEFSHGSNNGLKIAIDFCEEIKSKHPRITYADLYQLAGVVAVEVTGGPTVDFVPGRKDS  118
           ***:********************************************************

APX1_Lv    KISPKEGRLPNATKGAPHLRDIFYRMGLSDKDIVALSGGHTLGKAHADRSGFDGPWTREP  180
smAPX1     RISPKEGRLPNANKGVPHLRDIFYRMGLSDKDIVALSGGHTLGKAHADRSGFDGPWTSEP  178
           :***********.**.***************************************** **

APX1_Lv    LKFDNSYFVELLKGESEGLLKLPTDIALLDDPTFRPYVELYAKDEDAFFNDYATSHKKLS  240
smAPX1     LKFDNSYFVELLKGESEGLLKLPTDVALLEDPGFRHYVDLYAKDEDAFFADYAVSHKKLS  238
           *************************:***:** ** **:********** ***.******

APX1_Lv    ELGFTPSSTKSKVKDSVILAQSAVGVIVAAAVVIVSYVYEARKKMK      286
smAPX1     ELGFTPNSSSCKL-DSVVLAQSAFGVAVAAAVVVVGYIFESRKRMK      283
           ******.*:..*: ***:*****.** ******:*.*::*:**:**
```

Table S1: Evolution of the accumulation of SILM and its different constituents during *S. marianum* fruit maturation (in whole achenes (WA), pericarps (P) and seed (E) from stage 1 to stage 6 of maturation).

	Taxifolin	Silychristin	Silydianin	Silybin A	Silybin B	Isosilybin A	Isosilybin B	Silymarin	ABA
**WA1**	0.05±0.03	0.00±0.00	0.02±0.00	0.00±0.00	0.07±0.00	0.00±0.00	0.00±0.00	0.14±0.03	1.83±0.38
**WA2**	0.05±0.01	0.00±0.00	0.03±0.00	0.00±0.00	0.24±0.01	0.04±0.00	0.06±0.02	0.42±0.02	5.57±0.63
**WA3**	0.03±0.00	0.00±0.00	0.04±0.00	0.00±0.00	0.25±0.06	0.04±0.00	0.15±0.03	0.50±0.03	13.63±1.42
**WA4**	0.36±0.01	0.51±0.03	0.31±0.00	0.39±0.03	1.35±0.21	0.47±0.05	1.21±0.12	4.58±0.23	29.10±1.91
**WA5**	0.47±0.02	0.47±0.02	1.01±0.18	1.73±0.20	10.48±0.41	1.29±0.16	8.75±0.51	24.20±1.50	48.57±1.56
**WA6**	0.91±0.08	1.15±0.03	2.63±0.08	4.06±0.07	22.64±1.28	2.80±0.12	18.26±0.31	52.46±1.93	46.37±2.15
**P4**	0.14±0.01	0.00±0.00	6.00±0.00	0.00±0.00	24.16±0.32	0.00±0.00	0.00±0.00	24.85±0.40	18.20±1.89
**S4**	0.02±0.01	0.00±0.00	0.42±0.02	0.02±0.01	0.02±0.01	0.01±0.00	0.07±0.04	0.56±0.07	21.47±2.48
**P5**	0.71±0.22	0.00±0.00	1.66±0.11	3.80±2.19	128.36±17.32	8.66±1.27	6.89±1.03	150.08±17.77	24.03±3.83
**S5**	0.00±0.00	0.12±0.04	1.20±0.07	0.08±0.02	2.12±0.13	0.10±0.02	0.65±0.03	4.27±0.01	28.27±1.94
**P6**	1.44±0.07	1.18±0.15	0.77±0.01	12.58±1.37	72.44±0.12	5.29±0.11	9.58±0.30	103.27±1.50	25.50±2.17
**S6**	0.01±0.01	1.01±0.05	0.32±0.00	0.46±0.10	1.38±0.09	0.05±0.01	0.22±0.05	3.45±0.02	31.67±0.42

Values expressed in mg/g DW are means ± SD of 3 independent experiments.

Table S2: Pearson correlation coefficient between PAL, CHS, POX and LAC activities determined during *S. marianum*.

	PAL	CHS	POX	LAC
PAL				
CHS	0.969**			
POX	0.995***	0.969**		
LAC	-0.522	-0.444	-0.487	

** $p < 0.01$, *** $p < 0.001$

Table S3: Primers and characteristics of the genes used for gene references selection for RT-qPCR analysis in maturing fruit of *S. marianum*.

Gene description	Primer Sequence (Forward/Reverse Primer)	Amplicon length (bp)	Fusion temperature (°C)	Identity percentage of Arabidopsis	Arabidopsis orthologue	Contigs *Silybum marianum*
Actin (ACT)	5' -TTCAGGCTGTTCTTTCTCTG- 3'<br>5' -TGCTGGCCGTGACTTGACTG- 3'	155	82.06	87	NM 00133666	141335
Cyclophilin (CYC)	5' -TGATTGCGGTCAACTTTCTTAG- 3'<br>5' -CTTCATGAATCTTCGTTCATACATG- 3'	120	87.94	92	AK228231	175903
Elongation Factor 1-α (EF1A)	5' -GCATCCAACTTCACTTCTCA- 3'<br>5' -ACCAAGATTGACCGTAGGTC- 3'	140	91.76	85	AK226639	223085
Elongation Factor 2 (EF2)	5' -GTGGGGCCGAGATCGTGGT- 3'<br>5' -CCTAACAAACACAACCGTTT- 3'	109	88.37	75	NM 001338012	65194
Eukaryotic translation initiation Factor 1 (ETIF1)	5' -TCTCGTCGGACTTCGTGATT- 3'<br>5' -TTGTCGGTGGAATTGATGAT- 3'	145	74.15	81	NM 122959	64211
Eukaryotic translation initiation Factor 3E (ETIF3E)	5' -CTACTGCAGAATACACGAGC- 3'<br>5' -ATTACCTTTTAGGGTTCAAG- 3'	131	87.55	71	AYY735589	114663
Eukaryotic translation initiation Factor 3H (ETIF3H)	5' -AATCTTGAATTGAACTAAAGA- 3'<br>5' -TGAGATGTTTGAGGGAAAAC- 3'	166	80.65	90	NM 100960	201771
Eukaryotic translation initiation Factor 4E (ETIF4E)	5' -TGTGGTTTGAAACTCTGATG- 3'<br>5' - AATGAGGCAGCTCAGGTGTG- 3'	145	79.42	77	NM 203123	194119
Eukaryotic translation initiation Factor 5A (ETIF5A)	5' -TTCCACATGTTAATCGTACC- 3'<br>5' -TGGTTTTGCCGAGGGAAAG- 3'	158	82.84	78	NM 105608	7686
Glyceraldehyde 3-phosphate deshydroigenase (GAPDH)	5' -AGGTTCTGCCTGCGCTTAAT- 3'<br>5' -CAAGGCTGCTATCAAGTAAG- 3'	138	80.06	76	KX086568	101004
Ubiquitin (UBI)	5' -TTGAGAGGTGGTATGCAGAT- 3'<br>5' -CAAAGATCCAAGACAAGGAA- 3'	114	83.36	91	U84968	31518
Ubiquitin extension protein (UBI2)	5' -CAAGATCCAAGACTGAAGA- 3'<br>5' -AGTCAACTCTTCACCTTGTCC- 3'	174	74.11	88	NM 001202839	30458

Table S4: Primers and characteristics of the SILM biosynthetic genes and ABA biosynthetic and signalling genes used for gene expression by RT-qPCR analysis in maturing fruit of *S. marianum*.

Gene description	Primer Sequence (Forward/Reverse Primer) 5'→ 3'	Amplicon length (bp)	Fusion temperature (°C)	Identity percentage of Arabidopsis	Arabidopsis orthologue	Contigs *Silybum marianum*
Phenylalanine Ammonia Lyase (PAL)	TGGCACAAAGGATGGCCATT<br>GTAACCGGTCCCTGTCGATC	120	78.54	74	AT2G37040	224771
Cinnamyl Alcohol Dehydrogenase (CAD)	TCCTGCGAAAGTTGCACTAA<br>CCAACGGAGAACAAAATGCT	144	80.46	75	AT3G19450	162290
Chalcone isomerase (CHI)	TGGCACAAGCAGTAATCGAG<br>TGAGCACATGATTTTGCATTT	195	79.52	70	AT3G55120	51573+125439
Ascorbate Peroxidase 1 (smAPX1)	CACACAAGAAGCTCTCGGAAT<br>AATCCCGGAAGAGGATGAAG	152	81.55	73 (atAPX3)<br>67 (atAPX1)	AT4G35000<br>AT1G07890	177494
Ascorbate Peroxidase 1 (Lv17APX1)	TGTTGAGCTGTATGCCAAGG<br>CGAAGCCCGTAAAAAGATGA	206	81.39	73 (atAPX3)<br>64 (atAPX1)	AT4G35000<br>AT1G07890	Lv et al. 2017
ABA Deficient 1 (ABA1)	GTGGGCTGGTACTGGCTCTA<br>CGATAAGGGTGTGGCAAAAC	165	82.34	77	AT5G67030	Lv et al. 2017
Leafy Cotyledon 2 (LEC2)	AAAAGGCACGTGAAATGAGC<br>TTTTATGCCCCAAATAACGTG	153	79.52	67	AT1G28300	39718

Table S5: List of *cis*-acting elements located in the SILM biosynthetic gene promoter regions

ELEMENTS	SEQUENCES	PLACE NUMBER	ROLES
1. Tissue expression: Akene			
AACA Motif	AACAAAC	S000353	Minimal *cis*-element requirements for endosperm-specific gene expression (cereal glutenin box)
Cereal Glutenin Box	TGTAAAAGT	S000034	Sequence responsible for the seed specific promoter activity (pea legumin gene in tobacco, homolog to the glutenin box)
SEF1MOTIF	ATATTTAWW	S000006	Sequence found in 5′-upstream region of soybean β-conglicinin (7S globulin); SEF=soybean embryo factor
SEF3MOTIFGM	AACCCA	S000115	Sequence found in 5′-upstream region of soybean β-conglicinin (7S globulin); SEF=soybean embryo factor
SEF4MOTIFGM7S	RTTTTTR	S000103	Sequence found in 5′-upstream region of soybean β-conglicinin (7S globulin); SEF=soybean embryo factor
NAPINMOTIFBN	TACACAT	S000070	Sequence found in 5′ upstream region of napin (2S albumin) gene in *Brassica napus*; seed specific expression
DRE2COREZMRAB17	ACCGAC	S000402	Expression during late embryogenesis, and is induced by ABA and drought
ACGTCBOX	GACGTC	S000131	bZIP protein DNA binding site for seed specific expression
CANBNNAPA	CNAACAC	S000148	Embryo- and endosperm-specific transcription of napin (storage protein) gene; seed specificity
CAATBOX1	CAAT	S000028	"CAAT promoter consensus sequence" found in legA gene of pea for seed expression
PROLAMINBOXOSGLUB1	TGCAAAG	S000354	*cis*-Element requirements for endosperm-specific gene expression
GCN4OSGLUB1	TGAGTCA	S000277	Required for endosperm-specific expression
MYBPZM	CCWACC	S000179	Core of consensus maize P (MYB homolog) binding site (gene specifies red pigmentation of kernel pericarp)
2. Hormone signaling: ABA			
ABRELATERD1	ACGTG	S000414	ABRE (ABA responsive element)-like sequence
DPBFCOREDCDC3	ACACNNG	S000292	Sequence binding for ABI5 bZIP transcription factors (ABA response and embryo-specific expression)
EBOXBNNAPA	CANNTG	S000144	E-box of napA storage-protein gene of *B. napus* (ABA response and seed specific expression)
RYREPEATBNNAPA	CATGCA(T/Y)(G)	S000264	"RY repeat" required for ABI3-dependent response to ABA and seed specific expression
